# Sydenham Society Publications

**Published:** 1860-06

**Authors:** 


— Two volumes of the publications of the New Sydenham Society have recently been received, which make five volumes issued by this Society for the year 1859. We learn from the report of the Council, that there are over 2,000 subscribers to their publications, and the list is rapidly increasing. With an increase in the amount received from members, the frequency of the publications will increase. Should the list reach 3,000, the Council propose to issue, in addition to the works already agreed upon, an Atlas, containing three of Hebra's Illustrations of Skin Diseases. This latter work would be worth the price of the subscription alone, and will constitute, when completed, the best and most magnificent Atlas of the kind ever issued. Besides this, the publications for 1860 will be:

Clinical Memoirs on Abdominal Tumors and Intumescence, by Dr. Bright.

A Year Book for 1859: Being a Register in condensed Abstract of all important communications, whether British or Foreign, in Medicine, Surgery, and their allied Sciences during the year.

Frerich's Clinical Account of Diseases of the Liver.

Vogel and Neubauer on the Examination of the Urine.

The subscription price to this Society is but $5.25 a year. Dr. C. F. Heywood, No. 66 West Twentieth Street, is the Local Secretary for New York.

